# Validation of a software‐based torsional measurement method of the lower limb: A retrospective study

**DOI:** 10.1002/ksa.12509

**Published:** 2024-10-21

**Authors:** Leonard Grünwald, Shuntaro Nejima, Tina Histing, Steffen Schröter, Peter Hagedorn

**Affiliations:** ^1^ Department of Traumatology and Reconstructive Surgery, BG Trauma Center Tübingen Eberhard Karls University Tübingen Tübingen Germany; ^2^ Osteotomie Komitee der Deutschen Kniegesellschaft München Germany; ^3^ Yokohama City University School of Medicine Yokohama Japan; ^4^ Diakonie Klinikum GmbH Jung‐Stilling Krankenhaus Siegen Siegen Germany; ^5^ Klinikverbund Südwest, Kliniken Sindelfingen Sindelfingen Germany

**Keywords:** CT, femur, software‐based measurement, tibia, torsional malalignment

## Abstract

**Purpose:**

The aim of this study was to evaluate a software‐based measurement tool of computed tomography imaging to assess torsional alignment of the lower limb—usually used for patients with posttraumatic deformities and patellofemoral issues—and compare these measurements with manual measurements of two experienced raters.

**Methods:**

This study was a retrospective analysis of 58 lower limbs (47 patients, 20 men and 27 women) aged between 19 and 97 years. Inclusion criteria were the clinical indication for torsion measurement of the lower limb. Legs with incomplete imaging and age less than 18 years were excluded. Femoral and tibial torsion were measured separately. Torsional alignment was assessed software‐based at two time points by two raters. Rater one additionally assessed manual‐based measurement at two time points. The software used was mediCAD 3D Knee Version 2.5.33 (Hectec). Subsequently, intra‐ and inter‐rater reliability was calculated using the intraclass correlation coefficient (ICC). Validity testing was performed by means of precision, concordance correlation coefficient according to Lin (ccc) and Pearson correlation coefficient.

**Results:**

High intra‐rater reliability for software‐based as well as manual‐based evaluation of torsional alignment was found for the tibia as well as the femur (ICC ranging between 0.870 and 0.993). Inter‐rater reliability also showed highly significant results of both manual and software‐based measurements (ICC ranging between 0.851 and 0.993). For almost all comparisons (except the tibia left), software‐based measurements showed higher ICC scores above 0.9, and, therefore, classified as ‘excellent reliability’. For validity testing, correlation coefficients and precision showed very good correspondence of the measurements (all values > 0.9), without systematic deviations.

**Conclusions:**

Software‐based measurement of torsional alignment according to the measurement method developed by H.‐A. Waidelich proved to be a reliable and valid technique. Especially for inexperienced surgeons, software‐based measurement, therefore, might improve confidence in reliable medical decisions in diagnostics and treatment.

**Level of Evidence:**

Level III.

**Abbreviations**
AI: artificial intelligence
ccc: concordance correlation coefficient
CT: computed tomography
DICOM: digital imaging and communications in medicine
FDA: Food & Drug Administration
ICC: intraclass correlation coefficient
PACS: picture archiving and communication system

## INTRODUCTION

Torsional alignment of the lower extremity is important after a fracture of the femur and tibia [24], as well as in congenital deformities [2, 4]. Although the importance of the treatment of torsional malalignment is proven, underdetection and undertreatment of axial plane malalignment is one major challenge for a holistic treatment approach. These difficulties derive from challenges with the accuracy and reliability of both physical examination and imaging [7, 9, 18, 22]. Surgical procedures like de‐rotational osteotomies, which have an influence on patellofemoral tracking [2, 16] and anterior knee pain [24], depend critically on reliable torsional alignment measurement.

Computed tomography (CT) measurement technique in the axial plane is the gold standard [5, 6, 15, 23, 26]. Depending on the measurement method, different norm values are described [8]. All methods use anatomical landmarks to define lines and angles [18]. The practical problem is to select the correct axial slice and superimpose the images to measure the correct angles [13]. Digital imaging and communications in medicine (DICOM) viewers are not able to superimpose images. Therefore, it is difficult to measure the exact angle. However, some techniques—using the transfer of superimposed pictures directly in the picture archiving and communication system (PACS)—measure the angles more easily. Although these concepts were able to measure torsion in the CT scan, documentation (transfer in the PACS) is not possible due to incompatible interfaces (depending on the used software). The measurement according to Waidelich et al. [25] became generally accepted, due to its reproducibility and generated standard values [21]. However, for the analysis of cases, planning and medico‐legal reasons, documentation is mandatory. To minimise measurement biases due to individual differences, software‐based measurement tools, such as the software mediCAD 3D Knee (Hectec), are increasingly important. Different medical software to measure the alignment in the coronal plane are meanwhile available. Even the intra‐rater and inter‐rater reliability is already described and the high quality (inter‐rater and intra‐rater reliability) is proven [17]. To the knowledge of the authors, there is just one medical software (Food & Drug Administration [FDA] approved) to measure torsional alignment on the market available.

The purpose of this study is to assess the reliability and validity of this software‐based measurement tool in comparison to the established manual measurement method. We hypothesised that the intra‐ and inter‐rater reliability would be high. The software‐based assessment of torsional alignment is comparable with a manual evaluation of an experienced rater.

## MATERIALS AND METHODS

Institutional review ethical board approval of the study was obtained (18.11.2020, IRB number: 695/2020B02). The study was conducted in accordance with the Declaration of Helsinki (as revised in 2013).

### Study design

In total, *n* = 50 patients were retrospectively selected randomly from 2005 to 2019. Some patients received several torsional CTs during this period; a total of *n* = 58 torsional CTs were selected, which corresponds to 116 torsion lower extremity measurements (left and right limb).

Torsional measurements were analysed twice by rater 1 with manual evaluation as well as software‐based at two different time points. Rater 2 additionally analysed all measurements with the software‐based tool for time points 1 and 2. For torsional measurement, the medical software mediCAD 3D Knee Version 2.5.33 (Hectec) was used.

### Technical parameters of the CT image acquisition for torsion measurement of the lower limb

CT image acquisition was performed using a 128‐slice, single‐source CT (SOMATOM Definition Edge, Siemens Healthineers). Reference settings were set as follows: hip (100 kV, 20 mAs), knee (80 kV, 20 mAs) and ankle (80 kV, 10 mAs). According to the institutional standard operating procedures, all patients were examined in a standardised supine position, with feet first, and the feet fixed together. The examinations were planned on a coronal scout view based on prominent anatomical landmarks: At the hip, top of the femoral head to the upper margin of the lesser trochanter; at the knee, top of the patella to the middle of the fibular head; and at the ankle, 2 cm above the tibial plafond to the tip of the medial ankle. Since 2021, the published ultra‐low‐dose protocol was used [10, 11].

### Evaluation of the lower limb torsion—manual measurements

**Figure 1:**
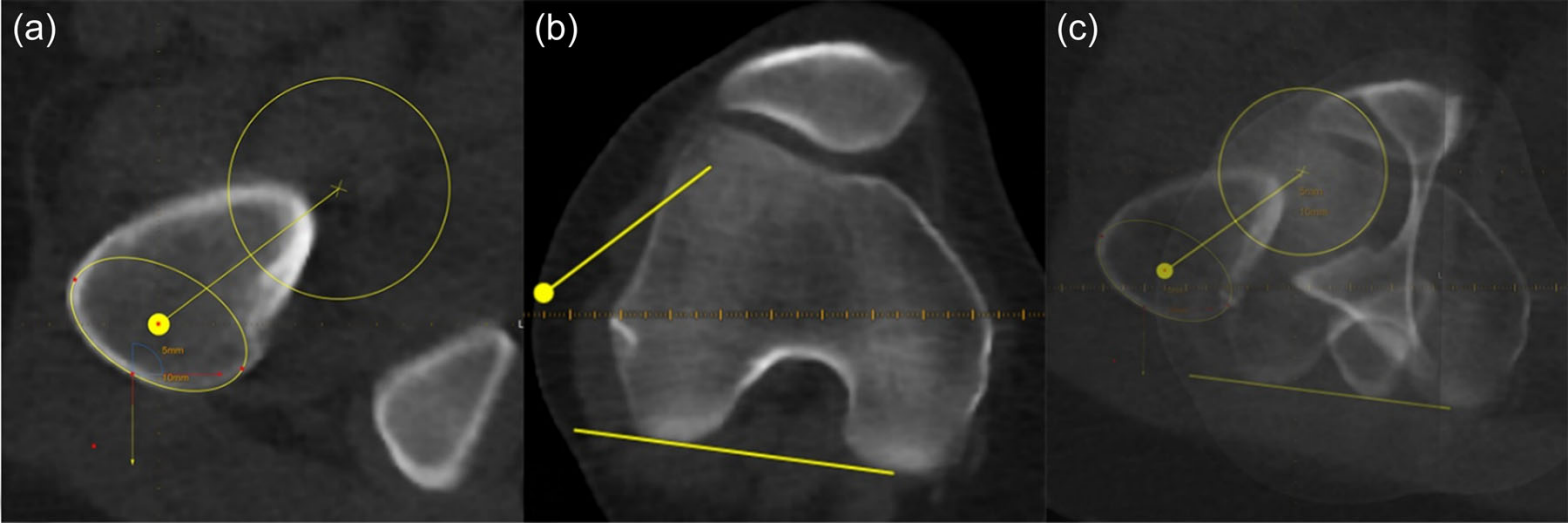
Landmarking (a) femoral head and major trochanter, (b) posterior condyles according to Waidelich and (c) superimposed image.

**Figure 2:**
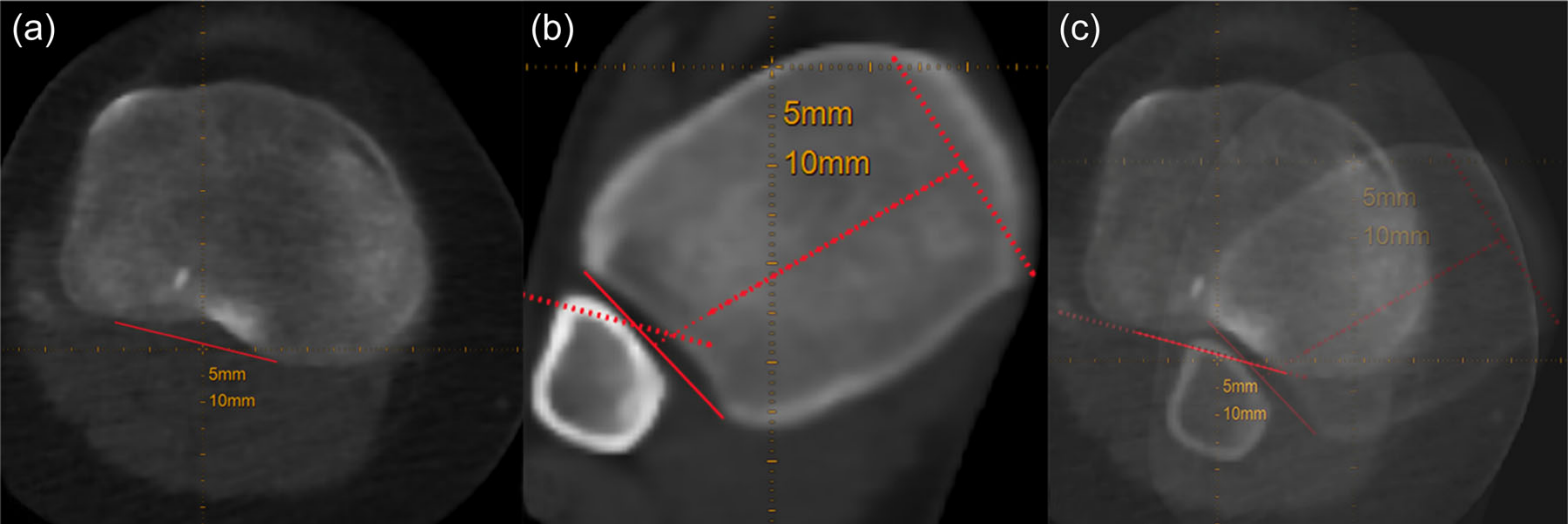
Landmarking (a) proximal tibia, (b) distal tibia and (c) superimposed image.

CT torsion measurements were performed on anonymised examinations and in randomised order using the programme Impax EE (Agfa HealthCare^®^). Femoral and tibial torsion were measured separately according to Waidelich et al. [25] femoral torsion was measured as the angle between a line central through the femoral head and the centre through an ellipse of the greater trochanter and a second line along the posterior margin (tangent) of the femoral condyles. The angle between these two lines of the centre of the hip and the centre of the trochanter and the tangent at the posterior margin of the femur condyles is the femoral torsion. Minus values describe internal torsion (distal in relation to proximal), and positive values describe external torsion (Figure 1). Tibial torsion was measured between a tangent along the posterior margin of the tibial plateau, and the distal tibial axis is defined as a line connecting the centre of the medial malleolus and the centre of the incisura fibularis (Figure 2).

### Evaluation of the lower limb torsion—software‐based measurements

Software‐based CT torsional measurement was performed using the FDA‐approved medical software mediCAD 3D Knee Version 2.5.33 module ‘torsion’. According to the defined workflow, the relevant CT slices of the hip, knee and ankle have to be connected by the software (‘stiching’). The semi‐automatic measurement for femoral and tibial torsion, according to Waidelich, can be selected. Anatomical landmarks were defined according to the workflow of the software. The landmarks of the software are the same as those used in Impax EE. However, software guides the user systematically through the whole measurements. Additionally, videos of each step are shown to support the user. These two videos are available in the supporting information on the journal's website.

**Figure 3:**
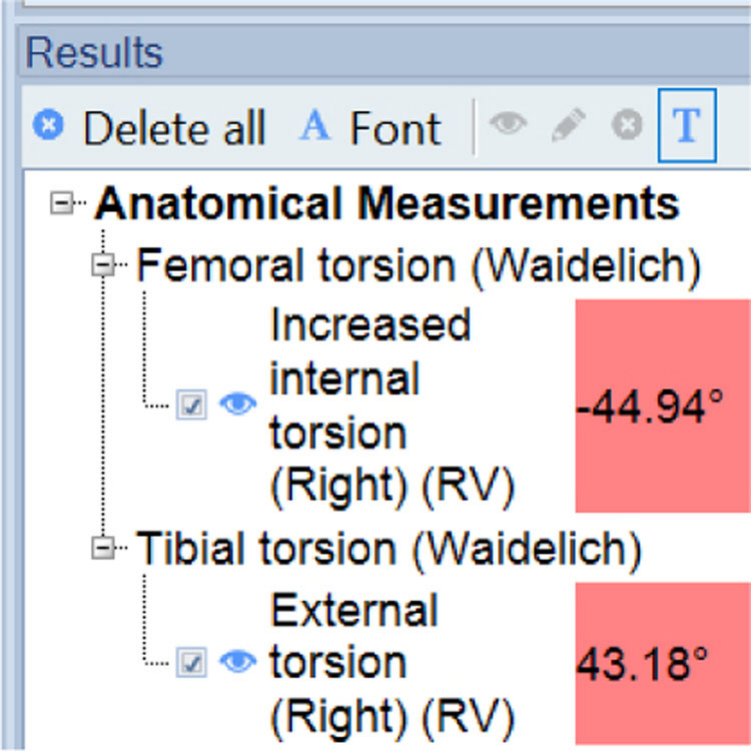
Results window of MediCAD Knee 3D.

After defining the landmarks, all defined angles are shown in the results window (Figure 3) and can be exported to the local PACS.

### Statistical analysis

For statistical analyses, IBM SPSS Statistics for Windows, version 27.0 (IBM Corp.) was used. To assess intra‐ and inter‐rater reliability, the intra‐class correlation coefficient was used. According to Bobak and colleagues, values between 0.75 and 0.9 indicate good reliability and any value above indicates excellent reliability [1]. Intra‐rater reliability was tested for the tibia and femur for manual measurement as well as software‐based measurement, conducted by rater 1. Inter‐rater reliability was tested for software‐based measurement—for tibia and femur each—by comparing rater 1 and rater 2 for both time points. For validity testing, the manual evaluation and the software‐based measurement were compared. Precision, Pearson correlation coefficient and the concordance correlation coefficient according to Lin (ccc) [12] were calculated for the femur and tibia each, for both time points of rater 1. The ccc is another method to compare different measurement methods with the benefit of detecting localisation and scale shifts. Values of 0 represent ‘no correlation’, and values of 1 represent ‘perfect compliance’. Bland–Altman plots were constructed afterwards. The level of significance was *α* ≤ 0.05.

## RESULTS

### Study sample

The sample size was planned, including a type I error rate of *α* = 0.01 and a type II error rate of *β* = 0.10 (i.e., Power = 0.90), assuming a bias of 0 and a correlation of *r* = 0.995 between the measurements. Both show a variability of 1.2 difference between the measurements and can be detected to be less than ±5 degrees with *n* = 47 observation pairs [14].

The total study sample was randomly selected out of 993 torsional CT scans and consists of *n* = 58 torsional CT scans of the lower limb from *n* = 47 patients (*n* = 20 male, *n* = 27 female). In 51.1% of the cases, the indication for the CT scan was chronic and congenital deformities combined with knee pain. 40.4% received the torsional CT scan after trauma, and for 8.5% of the cases, a combined problem, chronic and posttraumatic, was documented. Only legs with incomplete imaging and age less than 18 were excluded. One femur had to be excluded, due to imaging issues of the lesser trochanter. For all 58 lower limbs, descriptive data are displayed in Table 1.

**Table 1:** Measurement for all *N* = 58 lower limbs for femur and tibia at both time points for both measurement methods and both raters.

	t1				t2			
	femur		tibia		femur		tibia	
	*N* = 57		*N* = 58		*N* = 57		*N* = 58	
	Right	Left	Right	Left	Right	Left	Right	Left
Rater 1—Manual								
Minimum	−46.00	−56.30	21.40	14.10	−47.70	−59.30	23.40	14.00
Maximum	7.80	22.40	63.30	56.50	3.50	20.20	67.10	59.70
Mean value	−22.99	−25.46	37.11	34.87	−23.72	−26.32	38.34	34.79
Standard deviation	10.46	13.33	9.19	9.69	10.30	13.07	9.19	9.56
Rater 1—Software								
Minimum	−55.85	−58.01	22.13	13.50	−53.74	−57.95	21.71	16.63
Maximum	7.29	16.38	66.10	54.27	6.07	14.98	67.74	59.21
Mean value	−24.61	−26.17	38.70	34.90	−25.56	−26.92	38.50	36.01
Standard deviation	11.53	13.21	9.67	8.90	11.23	13.19	9.89	9.40
Rater 2—Software								
Minimum	−55.23	−58.97	21.88	12.78	−54.55	−59.23	20.72	12.69
Maximum	7.49	13.12	65.03	58.77	3.59	10.69	66.75	54.24
Mean value	−25.51	−27.44	37.60	32.90	−25.82	−27.55	36.40	33.30
Standard deviation	10.88	13.15	9.37	9.23	10.89	12.79	9.26	9.19

### Intra‐rater reliability

The period of time between measurements 1 and 2 was 3 weeks. The range of the results for the ICC was 0.870–0.970 (Table 2).

**Table 2:** Intra‐rater reliability for tibia and femur for both methods (rater 1) (intra class coefficient [ICC] and confidence interval [CI]).

	ICC between measurement 1 and 2	
Femur	Right	Left
Manual	0.964 [0.939, 0.979]	0.870 [0.786, 0.923]
Software‐based	0.990 [0.983, 0.994]	0.993 [0.988, 0.996]
Tibia		
Manual	0.952 [0.920, 0.971]	0.970 [0.950, 0.982]
Software‐based	0.962 [0.937, 0.977]	0.953 [0.922, 0.972]

### Inter‐rater reliability

Results for inter‐rater reliability ranged between 0.909 and 0.993 (Table 3).

**Table 3:** Inter‐rater correlation for tibia and femur for software‐based measurement (rater 1 + rater 2) (intra‐class coefficient [ICC] and confidence interval [CI]).

	ICC between rater 1 and 2	
Femur	Right	Left
Time point 1	0.985 [0.975, 0.991]	0.948 [0.914, 0.969]
Time point 2	0.993 [0.988, 0.996]	*r* = 0.851 [0.761, 0.910]
Tibia		
Time point 1	0.929 [0.883, 0.957]	0.918 [0.865, 0.951]
Time point 2	0.909 [0.851, 0.945]	0.955 [0.926, 0.973]

### Validity testing

Concerning validity testing, the range of all tested coefficients was *r* = 0.944 to *r* = 0.986 and was highly significant (Table 4).

**Table 4:** Pearson correlation (*r*) and concordance correlation coefficient according to Lin (ccc) for tibial and femoral measurement for time point 1 and 2 for rater 1.

	Femur				Tibia			
Time point	1		2		1		2	
	Right	Left	Right	Left	Right	Left	Right	Left
Pearson correlation	*r* = 0.974 *p* ≤ 0.001	*r* = 0.986 *p* ≤ .0001	*r* = 0.975 *p* ≤ 0.001	*r* = 0.981 *p* ≤ 0.001	*r* = 0.943 *p* ≤ 0.001	*r* = 0.956 *p* ≤ 0.001	*r* = 0.947 *p* ≤ 0.001	*r* = 0.965 *p* ≤ 0.001
Lin's ccc	0.959 *p* ≤ 0.001	0.985 *p* ≤ 0.001	0.957 *p* ≤ 0.001	0.980 *p* ≤ 0.001	0.985 *p* ≤ 0.001	0.953 *p* ≤ 0.001	0.944 *p* ≤ 0.001	0.957 *p* ≤ 0.001
Precision	0.984 *p* ≤ 0.001	0.999 *p* ≤ 0.001	0.982 *p* ≤ 0.001	0.999 *p* ≤ 0.001	0.999 *p* ≤ 0.001	0.997 *p* ≤ 0.001	0.997 *p* ≤ 0.001	0.992 *p* ≤ 0.001

**Figure 4:**
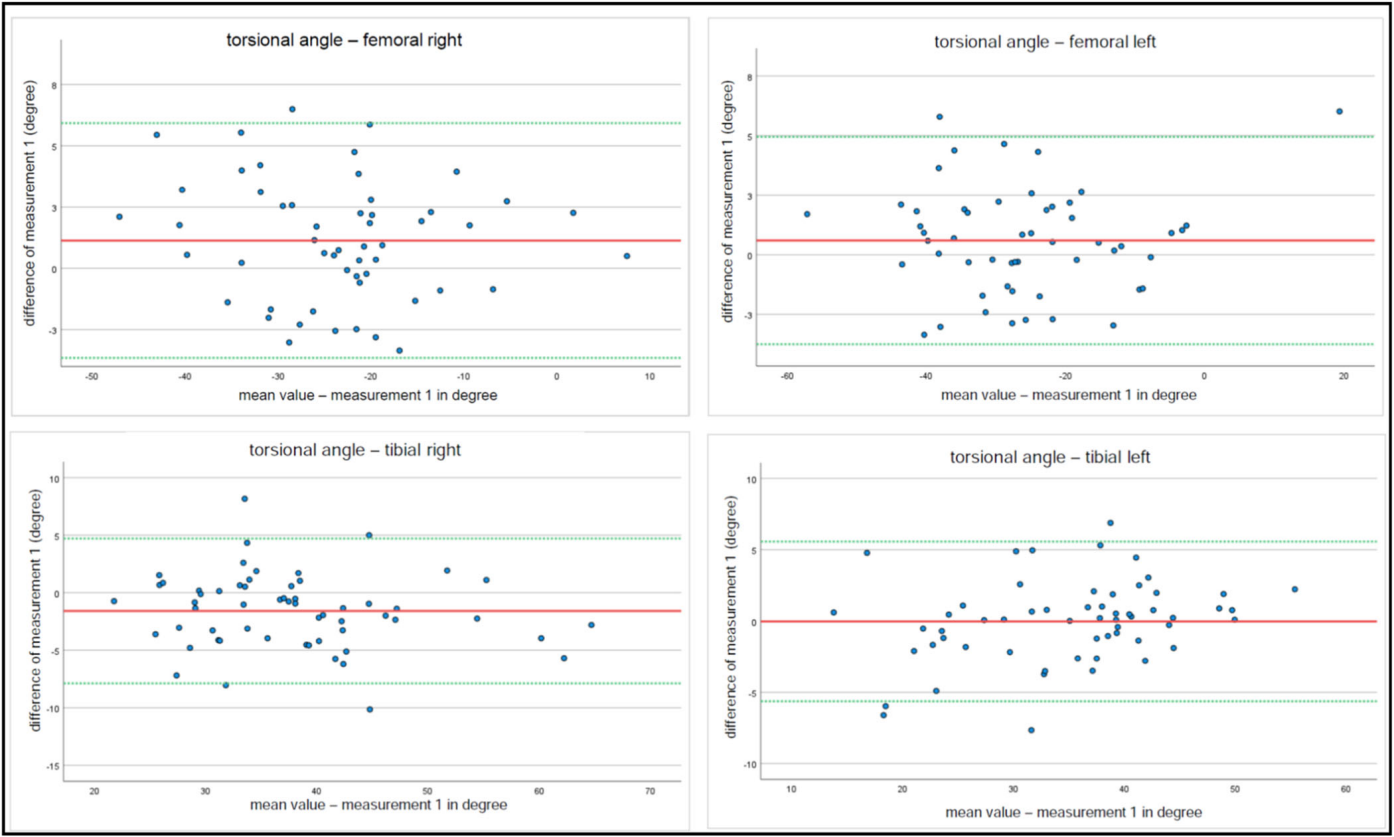
Bland–Altman Plots for average differences for manual measurements and software‐based measurements for time point 1.

**Figure 5:**
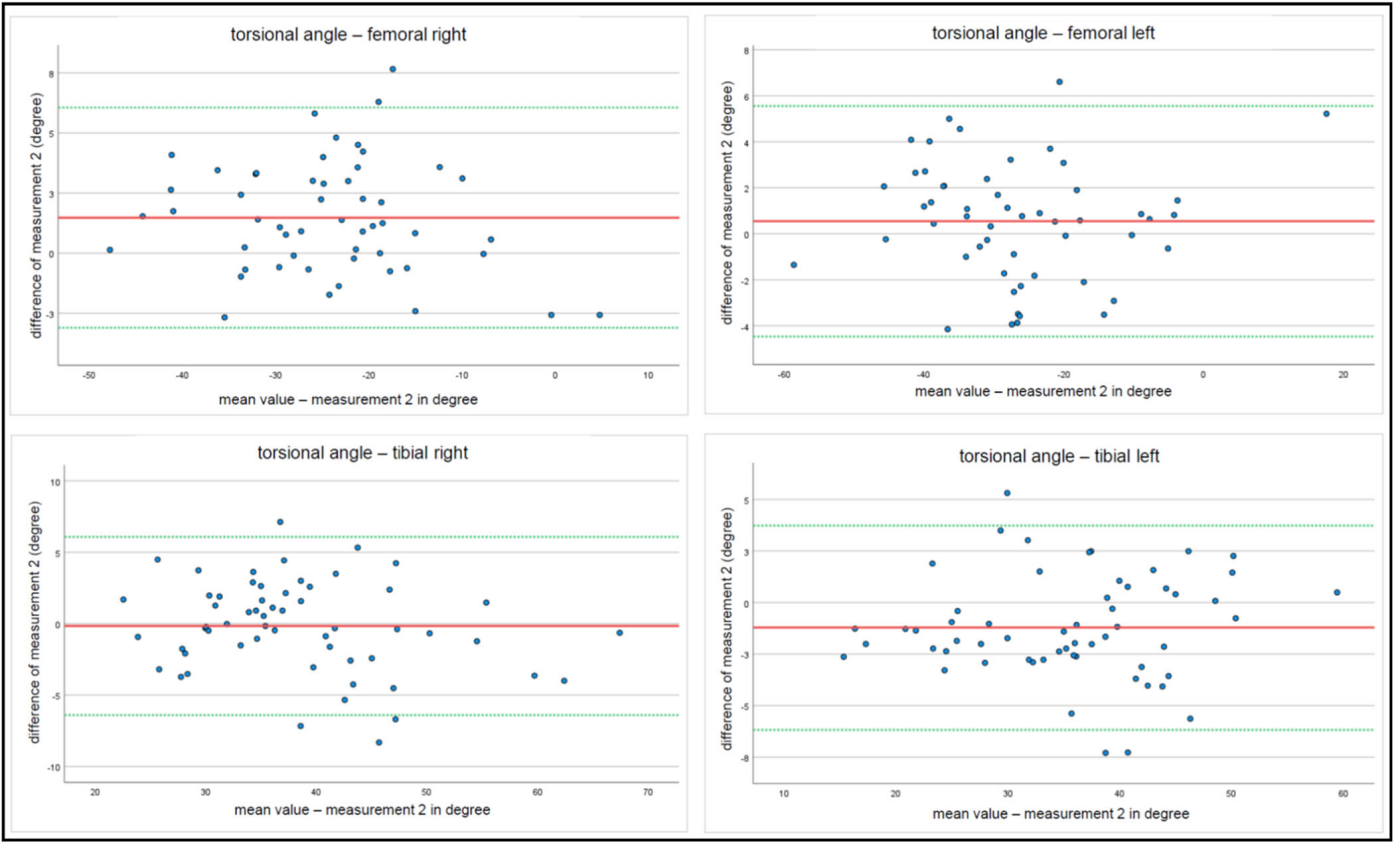
Bland–Altman Plots for average differences for manual measurements and software‐based measurements for time point 2.

To continue and develop the results further, Bland–Altman plots were generated. Average differences for manual measurements and software‐based measurements are displayed for time point 1 and time point 2 (Figures 4 and 5).

## DISCUSSION

The most important finding of the study was that the medical software‐based measurement of torsional alignment, according to Waidelich, was proven to be a reliable and valid method. High intra‐ and inter‐rater reliability for medical software‐based measurement, as well as manual‐based evaluation of torsional alignment, was demonstrated. Inter‐rater reliability between rater 1 and rater 2 also showed high concordance for ICC values of both manual and software‐based measurements. Femoral measurements showed slightly higher values in ICC than tibial measurements. For validity testing, correlation coefficients, precision and Bland–Altman plots showed good correspondence of the measurements. As for reliability testing, femoral comparisons were slightly more precise than tibial comparisons. These results are in line with the study of Liodakis et al. [13], who reported superior values for femoral compared to tibial measurements in the manual measurement method. Only for the bimalleolus method, tibial measurements of intra‐rater reliability showed better results. However, for these specific measurement methods, there are no norm values available [13]. For the values of this study, almost all correspond closely to the generated norm values of Strecker et al. [20].

The results of high intra‐ and inter‐reliability for comparison of measurements with Impax EE and the Waidelich method are in line with the study of Kaiser et al. [8], who found almost equal high results for inter‐ and intra‐rater reliability in their study using 26 cadaveric femora. Regarding medical software‐based alignment measurements, Schröter et al. examined the reliability of two medical software‐based measurement tools (PreOPlan [Siemens, Deutschland/Synthes, Schweiz], mediCAD version 2.20) in 81 radiographs for coronal alignment [17]. Alike to the study of Schröter et al. [17], Sled and colleagues tested the reliability of the software‐based programme Horizon Surveyor (Version 1.5, OAISYS Inc.) with 100 whole‐leg radiographs [19]. For software‐based measurements, good reliability with ICC values resulted between 0.839 and 0.998. These results are in line with the results of this study.

As a limitation of the presented results, it has to be stated that for the present sample, all patients were included, regardless if they had suffered from traumatic or congenital deformities. However, this sample represents exactly the patient collective, which usually relies on torsional alignment measurement. Therefore, it seems useful to conduct statistical testing with this patient subgroup to evaluate if measurements are stable in these special conditions. Nevertheless, it is advisable to develop standard values with a random sample of society.

Further development could also be automated programmes or artificial intelligence (AI) that assist orthopaedic surgeons in torsion measurement. There is a wide field of scientific research concerning the integration of AI use in medicine. The advantages are obvious, concerning a substantial time gain, which might relieve the medical system as a whole. Additionally, a precise working AI could also assist inexperienced colleagues in correct decision‐making and setting up treatment schedules. However, science is only in the beginning. For medical fields, such as pathology or cancer detection, there are already programmes with digital image algorithms available (i.e., navify Digital Pathology, Roche and Fraunhofer Institute, National Centrum für Cancer Diseases (NCT) Heidelberg). For orthopaedics, however, research is in the early beginning. In 2022, Erne and colleagues developed an AI‐based automated measurement tool for long‐leg radiographs with good correspondence to manual measurements [3]. However, this assessment of long‐leg radiographs has always been highly standardised and, therefore, might have been easier to adapt to an automated AI process. Nevertheless, this should be another important step in the measurement of torsional alignment.

## CONCLUSION

Software‐based measurement of torsional alignment is a beneficial and time‐saving tool to assist orthopaedic surgeons in diagnostics and treatment decisions, especially if they are inexperienced in the assessment of torsional alignment.

## AUTHOR CONTRIBUTIONS

**Grünwald Leonard**: Study protocol; data collection; statistical analysis; drafting and writing the manuscript. **Histing Tina**: Supervision and revising the manuscript. **Nejima Shuntaro**: Statistical analysis; review of the manuscript. **Steffen Schröter**: Study protocol; data collection; drafting and writing the manuscript. **Peter Hagedorn**: Study protocol; data collection and statistical analysis.

## CONFLICT OF INTEREST STATEMENT

The authors declare no conflict of interest.

## ETHICS STATEMENT

All procedures performed in the study involving human participants were in accordance with the ethical standards of the institutional and/or national research committee and with the 1964 Helsinki Declaration and its later amendments or comparable ethical standards. Institutional review board approval about all aspects of the study from an ethical and legal point of view was obtained (18.11.2020, IRB number: 695/2020B02). Due to the retrospective approach, no patient contact occurred in this study and informed consent was not needed to be obtained.

## Supporting information

Supporting Information.

Supporting Information.

## Data Availability

Data that support the findings of this study are available from the corresponding author, Grünwald Leonard, upon reasonable request.
